# Research and Remedies

**Published:** 1912-11-02

**Authors:** 


### RESEARCH AND REMEDIES.

### The Treatment of Ringworm.

SOME time ago publicity was given in these columns (THE HOSPITAL, February 24, 1912) to the new methods of treating ringworm which have been introduced this century, and to certain objections to them advanced by various observers. Dr. J. H. Sequeira now defends the *x*-ray treatment, which Dr. Riddell attacked last February in a pamphlet published for the Medical Officers of Schools Association (J. and A. Churchill, one shilling net). Dr. Sequeira's experience at the London Hospital extends to more than 2,500 cases treated by *x*-rays. Before the pastille method of measuring the dose was adopted the percentage of permanent baldness ensuing after the treatment was under 1 per cent., since that method has been used the percentage has fallen to 0.013. Dr. Sequeira prefers *x*-rays to ionisation; and lays stress on the fact that they merely cause epilation, and that therefore a mild parasiticide must be used to avert the possibility of re-infection.

### Circumcision in Girls.

A WELL-KNOWN and eminent New York surgeon, Dr. Robert Morris, commits himself in the *International Journal of Surgery* to advocacy of this operation on a fairly extensive scale. He says that one of the stigmata of decadence in the human female, is lack of full development of the clitoris with adherent prepuce. In some cases this is of no significance and causes no symptoms; but in others it gives rise to the same troubles as those which attend adherent prepuce in the male. Dr. Morris proceeds to state that girls require circumcision as often as boys do, and for the same general reasons. It does not appear in what percentage of boys Dr. Morris considers this operation advisable; but if his statements quoted above are correct, it is clear that there must be a great deal of decadence among American women. He describes the operation, which requires general anæsthesia; mere separation of adhesions does not suffice. It is not likely that this suggestion will be much acted on in Britain; but we quote it because its originator is a prominent man in the surgical world of New York.

### Olive Oil for Operative Shock.

DR. R. H. FERGUSON'S name is well known in this country as the pioneer of the open ether method of anæsthesia. In the *Long Island Medical Journal* he describes a proceeding which he holds to be of the greatest value for those who have been anæsthetised for an operation. This is the injection of six ounces of pure olive oil at a temperature of 104° F. into the sigmoid flexure, or higher, *via* the rectum. The benefit of this is explained to be that it maintains the activity of the phagocytes at the pre-anæsthetic level; whereas otherwise the ether or chloroform administered reduces it to vanishing point for several days. When the olive oil is given, phagocytosis regains the normal in three to five hours, according to Dr. Ferguson. Stress is laid upon the fact that pure olive oil alone is effective. Cotton-seed oil is not nearly so good, and mineral oils are useless altogether. Fuller details of this novel idea will be found in the *New York Medical Journal* of May 11, 1912.

